# Supplementary figures and images for: Investigating the ability of astrocytes to drive neural network synchrony
Source: PLoS Comput Biol. 2023 Aug 9;19(8):e1011290. doi: 10.1371/journal.pcbi.1011290 (PMC10441806; doi:10.1371/journal.pcbi.1011290)

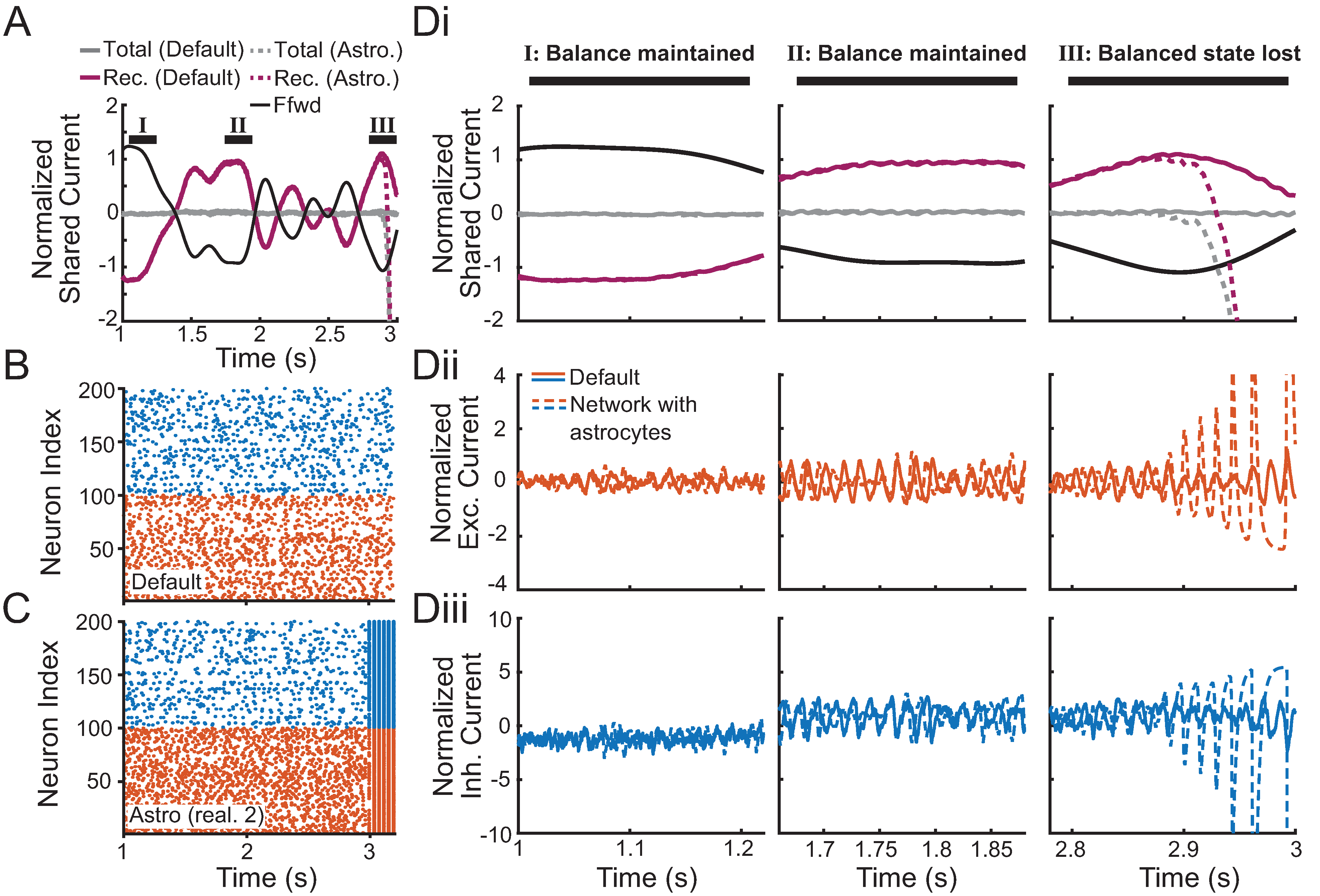

Supplement: S1 Fig — A: Normalized shared fluctuations in the feedforward (black) and recurrent (purple) synaptic inputs for two one population, non-spatial networks (solid: default; dashed: realization 2 from Fig 3C). B,C: Spiking times of sampled excitatory (red) and inhibitory (blue) neurons from the default network and network with astrocyte ensheathment, respectively. D: Normalized shared fluctuations in the currents zoomed in on time windows when the asynchronous state is maintained in both networks (left and middle columns) and for the time window when the asynchronous state is lost in the network with astrocyte ensheathment (right column). The following currents are displayed: i) feedforward, all recurrent, total, ii) recurrent excitatory, and iii) recurrent inhibitory currents in the default network (solid) and the network with astrocyte ensheathment (dashed). Same parameters as Figs 3 and 4. (TIF) [file pcbi.1011290.s001.tif]

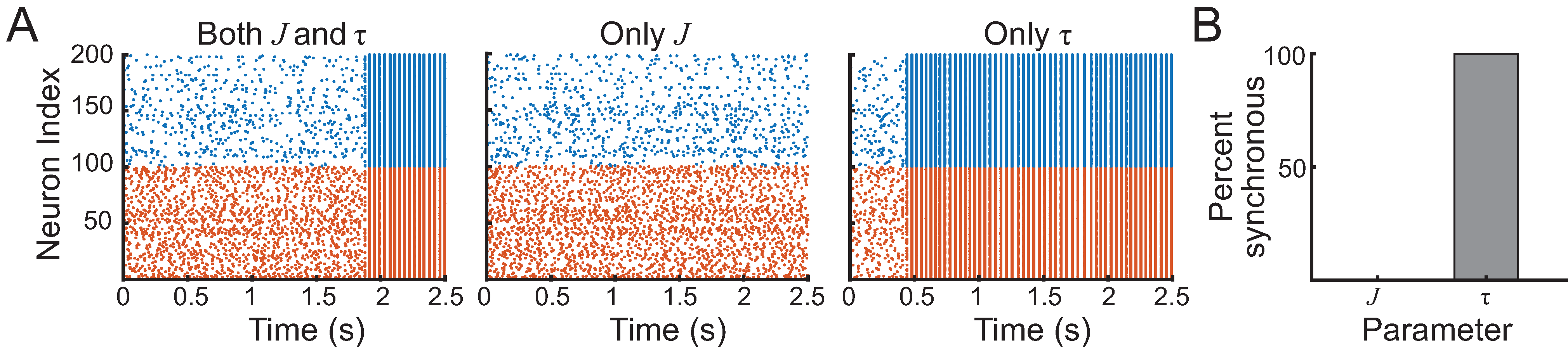

Supplement: S2 Fig — A: Spiking times of sampled excitatory (red) and inhibitory (blue) neurons from the one population, non-spatial network with astrocyte ensheathment tuning both the synaptic strength (J) and time constant (τ) (left), only J (middle) and only τ (right). B: Percent of ten networks that maintained synchronous activity when only J or τ were modulated by astrocyte ensheathment. Ensheathment parameters for all panels: sen = 0.4, pene=0.7 and peni=0. (TIF) [file pcbi.1011290.s002.tif]

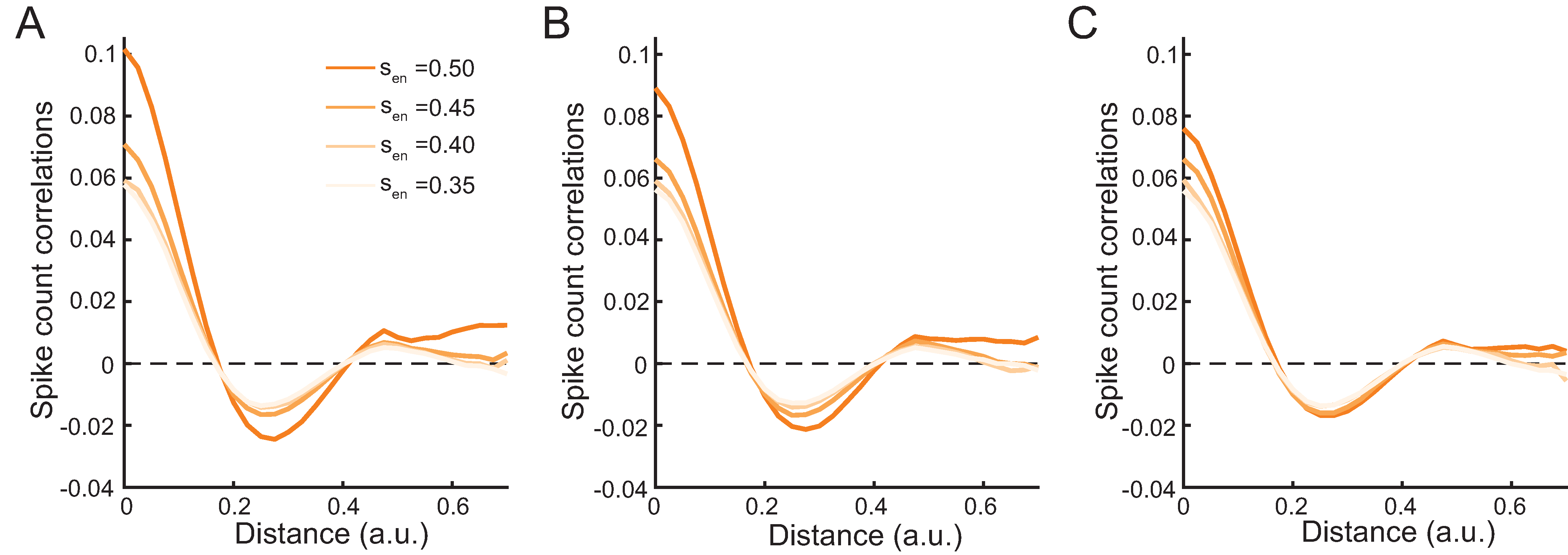

Supplement: S3 Fig — Spike count correlations as a function of distance for different probabilities of ensheathment (A: pene=0.9, B: pene=0.85, C: pene=0.8) and ensheathment strengths (sen ∈ [0.35, 0.5]), where the default network exhibits spatial correlations (αrec = 0.2 and αffwd = 0.1). (TIF) [file pcbi.1011290.s003.tif]
